# Copper compound induces autophagy and apoptosis of glioma cells by reactive oxygen species and jnk activation

**DOI:** 10.1186/1471-2407-12-156

**Published:** 2012-04-27

**Authors:** Cristina Trejo-Solís, Dolores Jimenez-Farfan, Sara Rodriguez-Enriquez, Francisca Fernandez-Valverde, Arturo Cruz-Salgado, Lena Ruiz-Azuara, Julio Sotelo

**Affiliations:** 1 Departamento de Neuroinmunología y Neuropatología, Instituto Nacional de Neurología y Neurocirugía, SSA, Insurgentes Sur 3877, México, DF, 14269, Mexico; 2 Facultad de Odontología, Universidad Nacional Autónoma de México, México, DF, Mexico; 3 Departamento de Bioquímica, Instituto Nacional de Cardiología, SSA, México, DF, Mexico; 4 Departamento de Química y Medicina Nuclear, Universidad Nacional Autónoma de México, México, DF, Mexico

**Keywords:** Copper compounds, Autophagy, Apoptosis, ROS, NK, Casiopeinas

## Abstract

**Background:**

Glioblastoma multiforme (GBM) is the most aggressive of the primary brain tumors, with a grim prognosis despite intensive treatment. In the past decades, progress in research has not significantly increased overall survival rate.

**Methods:**

The in vitro antineoplastic effect and mechanism of action of Casiopeina III-ia (Cas III-ia), a copper compound, on rat malignant glioma C6 cells was investigated.

**Results:**

Cas III-ia significantly inhibited cell proliferation, inducing autophagy and apoptosis, which correlated with the formation of autophagic vacuoles, overexpression of LC3, Beclin 1, Atg 7, Bax and Bid proteins. A decrease was detected in the mitochondrial membrane potential and in the activity of caspase 3 and 8, together with the generation of intracellular reactive oxygen species (ROS) and increased activity of c-jun NH_2_-terminal kinase (JNK). The presence of 3-methyladenine (as selective autophagy inhibitor) increased the antineoplastic effect of Cas III-ia, while Z-VAD-*FMK* only showed partial protection from the antineoplastic effect induced by Cas III-ia, and ROS antioxidants (N-acetylcysteine) decreased apoptosis, autophagy and JNK activity. Moreover, the JNK –specific inhibitor SP600125 prevented Cas III-ia-induced cell death.

**Conclusions:**

Our data suggest that Cas III-ia induces cell death by autophagy and apoptosis, in part due to the activation of ROS –dependent JNK signaling. These findings support further studies of Cas III-ia as candidate for treatment of human malignant glioma.

## Background

Glioma is the most common primary brain tumor in humans [1]. Glioblastoma, a highly malignant glioma shows extensive angiogenesis, essential for tumoral growth and invasiveness. Its prognosis is poor and has not changed substantially in recent decades. Malignant gliomas are usually resistant to immunotherapy, chemotherapy, radiotherapy, and other adjuvant therapies [2]. In addition, glioma cells are prone to develop resistance to initially effective therapies. Cisplatin and carboplatin are used as first-line chemotherapy; however, significant myelosuppression (carboplatin) and nephrotoxicity (cisplatin) have limited their use [3]. Therefore, it is clinically relevant to identify novel chemotherapeutic agents against malignant glioma, ideally; they should show less collateral effects and better antitumoral activity.

In the search for new anticancer agents with effective chemotherapeutic spectrum and reduced toxicity, new substances based on endogenous (essential) metals have shown promising initial results. Recently, a group of copper-coordinated complexes have been developed (Casiopeinas®, Cu(N-N)(A-A)]NO_3_, (A-A = N-O, O-O)) with perceptible antineoplastic effects on human ovarian carcinoma (CH1), murine leukemia (L1210), AS-30D rat hepatoma, cervico-uterine (HeLa) and colon (HCT40) carcinomas [4-7].

Two major routes of programmed cell death (PCD) have been proposed. Type I, or apoptotic cell death, is a highly controlled process involving several well-characterized morphological changes, including cell volume loss, chromatic condensation and nuclear fragmentation [8]. Apoptosis induction may involve either extracellular triggering signals (such as tumor necrosis factor) or endogenous signals (such as a cytochrome c release) [9], followed by the activation of caspases and endonucleases [10]. This leads to disassembly of nuclear chromatin and degradation of oligonucleosomal DNA. Type II, autophagy, is a dynamic process involving the sequestration of cytoplasmatic portions and intracellular organelles into vacuoles called autophagosomes. These vesicles are fused with lysosomes to generate autophagolysosomes and mature lysosomes, where the sequestered material is degraded, leading to cell death [11].

Among the effector mechanisms involved in the control and regulation of cell death pathways, including autophagy and apoptosis, is the cellular redox status. The redox status in the cell is determined by the balance between the rates of production and breakdown of reactive oxygen and/or nitrogen species (ROS/RNS), including free radicals such as superoxide (O_2_^.-^), hydroxyl radical (HO^.^), and non-radicals capable of generating free radicals (i.e., H_2_O_2_) [12]. In previous studies, another copper compound (Cas IIgly [Cu(4,7-dimethyl-1,10-phenanthroline)(glycine)(H_2_O)]NO_3_) was shown to induce a dramatic drop in intracellular levels of reduced glutathione (GSH) in human lung cancer H157 and in A547 cells. GSH was used as a source of electrons to catalyze the Fenton reaction leading to ROS formation and cell death [13]. ROS are regulators of mitogen-activated protein kinase (MAPKS), a family of serine/threonine kinases, which mediates intracellular signal transduction in response to different physiological stimuli and stressing conditions [14,15].

Three major MAPKs have been identified; c-jun NH_2_-terminal kinase (JNK), p38, and extracellular signal-regulated kinase (ERK 1/2). JNK and/or p38 activation induce apoptosis, while ERK activation favors proliferation and promotes cell survival [16,17]. An increase in intracellular ROS, as well as the activation of MAPKs, participate in autophagic execution [18,19].

Here we report the mechanisms of cell death induced in C6 glioma cells by the copper compound Casiopeina III-ia [Cu (4,4´ dimethyl-2,2 bipiridine) (acetoacetonate)] NO_3_ (Cas III-ia)_._ Exposure of C6 glioma cells to Cas III-ia resulted in cell death, with ultrastrucrural and biochemical features consistent with autophagy and apoptosis. Futhermore, the involvement of ROS generation and JNK activation as major features of the autophagic and apoptotic pathways was demonstrated.

## Methods

### Cas III-ia synthesis

Cas III-ia was synthesized as previously described (Ruiz-Ramírez et al. [4]) and dissolved in sterile water.

### Glioma cell culture

Rat glioma C6 cells (American Tissue Culture Collection, Rockville, Maryland, USA) were maintained at 37°C in 5% CO_2_ and 95% O_2_ under sterile conditions in Dulbecco modified Eagle medium (Sigma Chemical Co, St. Louis, MO USA), supplemented with 10% fetal bovine serum plus 10 mg streptomycin, 10,000 units pencillin and 25 μg amphotericin B per ml (Sigma Chemical Co, St. Louis, MO USA). After 24 h of culture, the glioma cell medium was replaced with fresh medium plus Cas III-ia (5, 10, 15 and 20 μg/ml).

The role of autophagy, apoptosis and ROS in the antineoplastic effect of CasIII-ia was examined by adding 5 mM of 3-methyladenine (3-MA; Sigma Chemical Co, St. Louis, MO USA) or 50 μM of benzycarbonyl-Val-Ala-Asp Z-VAD (Z-VAD-*FMK*; BD PharMingen, San Diego, CA USA) or 10 mM of *N*-acety-L- cysteine (NAC; Sigma Chemical Co, St. Louis, MO USA) or JNK inhibitor (SP600125; 25 μM, Calbiochem, San Diego, Ca USA) or ERK inhibitor (PD98059; 25 μM, San Diego, Ca USA) to the control and the Cas III-ia treated cells. As positive control for autophagy, C6 glioma cells were treated with 250, 500 and 1000 μM temozolamide (TMZ, Shering-Plough Research Institute, Kenilworth, NJ, USA) with or without 5 mM 3-MA for 24 h.

### Cell viability assay

Cells were plated in 96-well microtiter plates at a density of 5x10^4^ per well in medium. Twenty-four h later, cells were treated with Cas III-ia; untreated cells served as control. After treatment for 24 h, cell viability was assayed as described previously [20], using 3[4,5-dimethylthiazol-2-yl]-2,5-diphenyl-tetrazolium bromide (MTT; Roche Diagnostics, Mannheim, Germany).

### Transmission electron microscopy

Cells were harvested, pelleted and fixed in 2.5% glutaraldehyde/2% PFA in a cacodylate buffer. The samples were post-fixed with 2% osmium tetroxide for 1 h, rinsed with fresh water and dehydrated in a graded alcohol series (50%, 75% and 95-100%). Finally, the samples were kept overnight in 1:1 propylene oxide/PolyBed 812, embedded in Poly Bed 812 and cured at 60°C. Ultrathin sections (2-3 μm) were obtained with a Reichert ultracut S microtome (Leica Microsystems, Wetzlar, Germany). Sections were stained with 2% uranyl acetate and 0.3% lead citrate and photographed using a Joel 1200 EX11 Transmission Electron Microscopy (Leica Microsystems, Wetzlar, Germany) with an oil immersion Plan-Apochromat × 63/1.4 NA objective lens.

### Autophagy assay

Control and treated cells (5X10^5^) were cultured on 0.7 cm^2^ round glass coverslips fixed in 8-multiwell plates (Daigger, Vernon Hills, IL USA) and incubated with 50 nM LysoTracker Red (LTR; Molecular Probes, Eugene, OR USA) at 37ºC for 10 min, to detect lysosome formation [21]. Serial confocal images were visualized using a Zeiss LSM 510 inverted laser scanning confocal microscope (Carl Zeiss, Oberkochen, Germany). LTR excitation at 543 nm was provided by a helium/neon laser, and fluorescence emission was measured by a 560-nm long pass barrier filter.

### Immunofluorescence assay

For immunostaining, control and treated cells were cultured on 0.7 cm^2^ round glass coverslips in 8-multiwell plates (Daigger, Vernon Hills, IL USA). The cells were fixed with 4% PFA/PBS for 10 min, washed three times with fresh PBS, permeabilized with DAKO^®^ target retrieval solution (DakoCytomation, Carpinteria, CA USA) for 30 min at 95°C, and finally blocked with albumin at room temperature. Afterwards, cells were preincubated with the primary antibody (LC3; p-JNK or p-c-jun) at a final dilution of 1:100 (Santa Cruz Biotechnology, Santa Cruz, CA USA) for 30 min at room temperature and detected with rhodamine-conjugated or FITC-conjugated secondary antibody (Jackson ImmunoResearch Laboratories, West Grove, PA USA) at **1:200** final dilution. To visualize the fluorescence of the primary antibody, cells were exposed to 5 μg/ml DAPI (Vector Laboratories, Inc. Burlingame, CA USA) and registered with a Zeiss LSM 510 inverted laser scanning confocal microscope (Carl Zeiss, Oberkochen, Germany).

### Apoptosis determination

To assess apoptosis in C6 glioma cells after exposure to Cas III-ia we used the in situ Cell Death Detection Kit, with fluorescein (Roche Diagnostics, Mannheim, Germany). Apoptotic cells were visualized using a Zeiss LSM 510 inverted laser scanning confocal microscope (Carl Zeiss, Oberkochen, Germany). Cell death induction was monitored as the appearance of the Sub-G_0_ peak in cell cycle analysis. Briefly, control and treated cells (1X10^6^) were centrifuged and fixed overnight in 70% ethanol at 4°C; cells were washed, incubated for 1 h in the presence of 1 mg/ml RNAase A and 20 μg/ml propidium iodide at room temperature, and analyzed with a Becton Dickinson (San Jose, CA) FACScan flow cytometer.

### Mitochondrial transmembrane potential assay

Mitochondrial potential was determined by analyzing the mitochondrial retention of the cationic fluorescent dye rhodamine 123 (Rhod 123); [22]. Briefly, 1x10^5^ cells were treated with Cas III-ia for 24 h, washed with PBS and incubated with 20 μg/ml of Rhod 123 at 37°C for 20 min. Rhod 123-loaded cells were washed with fresh PBS to eliminate excess dye. Rhod 123 fluorescence was immediately measured with the FACSCalibur cytometer (Becton Dickinson, San Jose, CA USA). Data were analyzed with the Cellquest 3.1f analysis software (Becton Dickinson, San Jose, CA USA).

### Cytochrome c (cyt c) release assay

Control and treated glioma C6 cells were harvested and washed once with ice-cold PBS. The cells were then incubated with extraction buffer (10mM Hepes, 250 mM sucrose, 10 mM KCL, 1.5 mM MgCl_2,_ 1 mM EDTA, 1 mM EGTA, 0.05% digitonin, and 1mM phenyl-methylsulfonyl fluoride) at 4°C for 10 min. The supernatant containing the cytosol proteins was used for Western blot analysis of *cyt c*[23].

### Western blot

Samples (30 μg protein) were resolved to 10-15% SDS-PAGE and transferred to a nitrocellulose membrane. The membrane were subsequently blocked and incubated with the respective primary antibody at a **final dilution of 1:500** (**LC3**, Beclin, Atg 7, Bid, Bax; **cyt*c***, caspase 3, Caspase 8, p-JNK, JNK, p-ERK, ERK, c- jun, p-c-jun and β-actin (Santa Cruz Biotechnology, Santa Cruz, CA, USA) for 24 h at 4°C. Immunoreactivity was visualized by probing with a horseradish peroxidase-conjugated secondary antibody (Santa Cruz Biotechnology, Santa Cruz, CA USA) and detected using the ECL kit (Santa Cruz Biotechnology, Santa Cruz, CA USA).

### Measurement of ROS formation

DCFH-DA (2',7'-dichlorofluorescein diacetate) is a stable, non-fluorescent molecule, which is hydrolized by esterases to the non-fluorescent DCFH (2',7'-dichlorofluorescein). DCFH is oxidized in the presence of ROS (superoxide anion, hydrogen peroxide, and hydroxyl radicals) turning into the highly fluorescent 2,7-DCF [24]. For analysis of reactive oxygen species (ROS), the DCFH-DA probe was used as previously described [24]. Briefly, lysed cells were diluted at 1:10 with 40 mM Tris (pH 7.4) and loaded with 5 μM DCFH-DA (molecular probes) in methanol for 15 min at 37°C. Subsequently, fluorescence was measured both prior to and 60 min after incubation. The formation of the fluorescent oxidized derivative of DCFH, named DCF was monitored at an excitation wavelength of 525 nm (slit 5 nm). The bucket container was thermostatically maintained at 37°C. Autofluorescence of the cellular lysate was always below 6%. The fluorescent signals of both methanol (as vehicle) and substrates were recorded at the baseline, prior to the calculation of DCF formation, which was quantified using a standard curve (Sigma, Aldrich) in methanol. Analysis was done using a Perkin-Elmer LS50-B luminescence spectrometer**.**

### Superoxide dismutase activity

Total SOD activity in lysed cells was assayed as previously reported [25]. In brief, a competitive inhibition assay was performed using a xanthine-xanthine oxidase system to reduce NBT. The final content of the mixture reaction was: 0.122 mM EDTA, 30.6 μM NBT, 0.122 mM xanthine, 0.006% bovine serum albumin, and 49 mM sodium carbonate. Five hundred μL of lysed cells (1:50) were added to 2.45 mL of the mixture described above; then 50 μL of xanthine oxidase, at a final concentration of 2.8 U/L, were added and incubated in a water bath at 27°C for 15 min. The reaction was stopped with 1 mL of 0.8 mM cupric chloride and the optical density was read at 560 nm. One hundred percent of NBT reduction was obtained in a tube in which the sample was replaced by distilled water. To measure Mn-SOD activity, CuZn-SOD activity was inhibited with DDC [25]. Mn-SOD activity was assayed by incubating the sample with 50 mM DDC at 30°C for 1 h, which was then dialyzed for 3 h with 3 changes of 400 vol of 5 mM potassium phosphate buffer (pH 7.8)-0.1 mM EDTA. CuZn-SOD activity was obtained by subtracting the activity of the DDC-treated samples from the total SOD activity. One unit of SOD activity was defined as the amount of protein that inhibited NBT reduction by 50%. Results were expressed as U/mg protein*.*

### Catalase activity

CAT activity was determined as in the method described by Lowry [26]. In short, the supernatant (50 μL) was added to a quartz cuvette containing 2.95 mL of 19 mmol/L H_2_O_2_ solution prepared in potassium phosphate buffer (0.1mol/L, pH 7.4). The change in absorbance was monitored at 240 nm over a 5-min period using a spectrophotometer (Shimadzu UV-1201, Japan). Commercially available CAT was used as standard. CAT activity was expressed as U/g tissue.

### Statistical analysis

All in vitro studies were made in triplicate. Data from experiments were analyzed by one –way ANOVA followed by Tukey’s multiple comparison test. A *P* value of <0.05 was considered significant.

## Results

### Cas III-ia induced growth inhibition and changes related to apoptotic and non-apoptotic cell death

**Figure 1:**
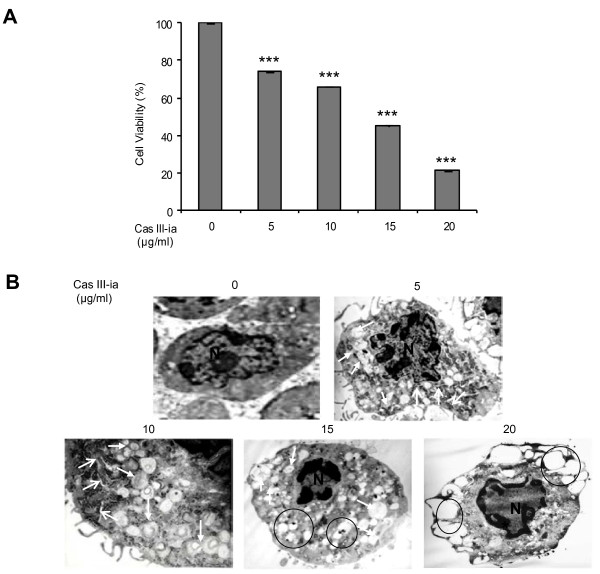
**Cas III-ia inhibits cell proliferation and induces morphologic changes in tumor cells. A)** Cell viability was determined by the MTT assay. Data represent the mean ± SD (*P ≤ 0.05, **P ≤ 0.01 and ***P ≤ 0.001) of three independent experiments. **B)** Electron micrograph untreated glioma C6 cells and magnified photographs of 24 h Cas III-ia-treated (5(1μm), 10 (500nm), 15(200nm) and 20 (1μm) μg/ml) glioma C6 cells. N indicates the nucleous, open white arrows point to endoplasmatic reticulum, filled thick arrow to autophagic vacuole, asterisk for margination and condensation of chromatin and black circles show numerous vacuoles observed by electronic microscope.

Exposure of C6 glioma cells during 24 h to increasing concentrations (5-20 μg/ml) of Cas III-ia resulted in a dose-dependent decrease of cell viability (Figure 1A). To investigate the mechanisms by which cell viability was reduced, ultrastructural changes were determined in C6 rat glioma cells treated with Cas III-ia for 24 h and examined by transmission electronic microscopy**.** At doses of 5 and 10 μg/ml of Cas III-ia, cells exhibited typical apoptosis-like nuclear morphology characterized by partial condensation and margination of chromatin along the nuclear envelope (Figure 1B); they also showed typical characteristics of autophagy: autophagic vacuoles delimited by a double-membrane, which contained cytoplasmic fragments. At the higher concentrations of 15 and 20 μg/ml of Cas III-ia, autophagic vacuoles contained disintegrated cellular structures, heavily vacuolized cytoplasm with a few short channels of endoplasmic reticulum, and nuclei with more condensed chromatin. These ultrastructural findings suggest the activation of both autophagic and apoptotic pathways***.***

### Cas III-ia induced death by autophagy

**Figure 2:**
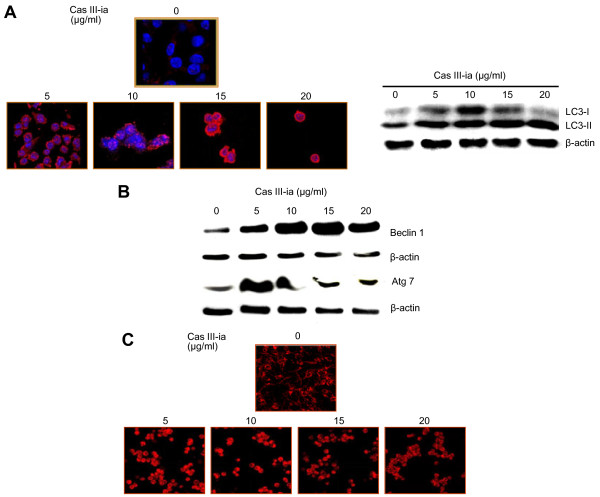
**The Cas III-ia induces autophagic marks. A)** Analysis of LC-3 (red-rodamine and blue–DAPI) by confocal microscope (left panel) and Western blot of LC-3 (right panel). **B)** Beclin 1 and Atg7 expression in cell lysates of control and Cas III-ia-treated glioma C6 cells were determined by Western blot. **C)** Accumulation of LysoTracker-red was analyzed in 24 h Cas III-ia-treated glioma C6 cells and controls by confocal microscope. The figures shown are representative of at least three different experiments for each experimental condition.

LC3 and Beclin 1 overexpression were evaluated in C6 glioma cells as indicative of autophagosomal activation [27]. The expression of LC3 was determined by immunofluorescence and Western blot. Analysis of control cells with confocal microscopy revealed the presence of a few red granules (LC3); in contrast, in Cas III-ia treated cells these red structures were more abundant (Figure 2A). Two forms of LC3 have been described: LC3-I and LC3-II (11). During formation of autophagosomes, the LC3-I cytoplasmatic form is cleaved and liquefied to give rise to the LC3-II membranous form. To determine which form of LC3 is affected by the presence of Cas III-ia, Western blot analysis was used to detected LC3-I and LC3-II levels. Results showed increased levels of LC3, particularly of LC3-II, leading to an increased ratio of LC3-II/LC3-I after Cas III-ia treatment (Figure 2A). Beclin 1 and Atg 7 expression were also determined by Western blot. All assayed doses of Cas III-ia treatment increased the expression of Beclin 1 and Atg 7 (Figure 2B). These results indicate that Cas III-ia induced autophagy promoters such as LC3-II, Beclin 1 and Atg 7.

To determine the effect of Cas III-ia on the activation of the lysosomal pathway, C6 glioma cells were loaded with LTR, which is a weak base that accumulates within the acidic lysosomal and autophagosomal compartments [21]. Confocal microscopy showed that, for all doses of Cas III-ia assayed, total LTR uptake increased as the lysosomal/autophagosomal compartment expanded, compared with control cells not exposed to Cas III-ia (Figure 2C). These results suggest that Cas III-ia induced autophagy in C6 glioma cells by the induction of Beclin 1 and Atg7 proteins and formation of autophagolysosomes.

Inhibition of Cas III-ia-induced autophagy enhances cell death in malignant glioma cells

**Figure 3:**
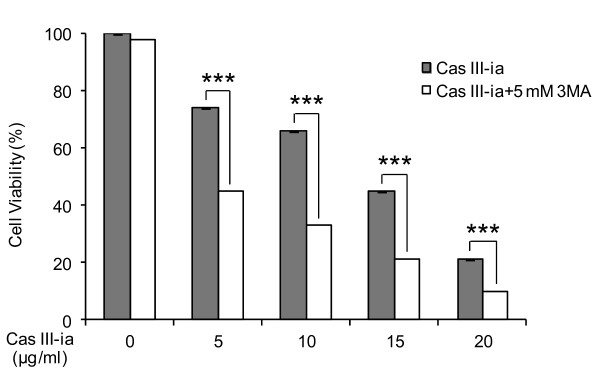
**3-MA enhanced cell death induced by Cas III-ia in C6 glioma cells.** A) Effects of 3-MA on Cas III-ia induced cytotoxicity were determined by the MTT assay in controls and cells treated with Cas III-ia and Cas III-ia + 5 mM 3-MA for 24 h; data represent the mean ± SD (*P ≤ 0.05, **P ≤ 0.01 and ***P ≤ 0.001) of three independent experiments.

To assess whether autophagy was induced by the Cas III-ia, the selective autophagy inhibitor 3-methyladenine (3-MA) was added to C6 glioma cultures. Treatment with 3-MA (5mM) alone had no significant effect on survival of C6 glioma cells. In contrast, the presence of 3-MA potentiated the decrease in cell viability induced by Cas III-ia treatment at all doses: from 74% to 45% at 5 μg/ml Cas III-ia, from 66% to 33% at 10 μg/ml, from 45% to 22% at 15 μg/ml, and from 21% to 10% at 20 μg/ml (Figure 3). These results demonstrated that cell death is enhanced in Cas III-ia-treated C6 glioma when autophagy is inhibited.

As positive control of autophagy, C6 glioma cells were treated with temozolamide (TMZ - 250, 500 and 1000 μM) with or without 3-MA (5 mM) for 24 h. TMZ inhibited cell viability in a dose-dependent manner. However, the presence of 3-MA significantly increased cell viability at all doses (Additional file l: Figure S1). TMZ, an alkylating agent, has been reported to inhibit cell viability of malignant glioma cells in a dose-dependent manner and to induce autophagy. When autophagy is subsequently prevented with 3-MA, localization of LC3 at the autophagosomal membrane is inhibited and tumor cells are rescued from cell death [28].

### Cas III-ia induced apoptosis

**Figure 4:**
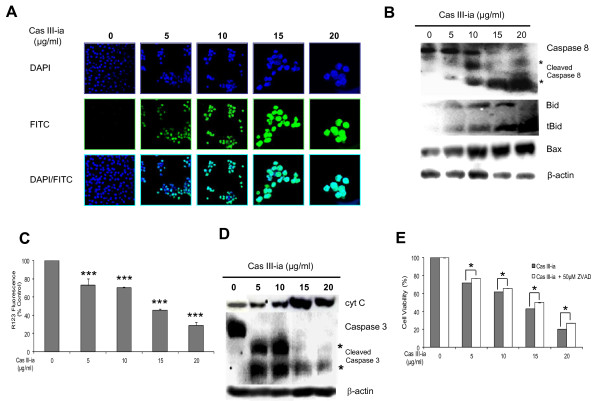
**Treatment with Cas III-ia induces apoptosis in tumor cells. A)** Apoptosis of control and 24 h Cas III-ia-treated glioma C6 cells (5, 10, 15 and 20 μg/ml) was determined by TUNEL assay and visualized by confocal microscope (Green-fluoresein and Blue-DAPI). The figures shown are representative of at least three different experiments for each experimental condition. Original magnification x20 for 5 and 10 μg/ml of Cas III-ia, and x40 for 15 and 20 μg/ml of Cas III-ia. **B)** Expression of capase-8, Bid and Bax proteins from controls and 24 h Cas III-ia–treated C6 glioma cells determined by Western blot. **C)** Mitochondrial membrane potential from controls and 24 h Cas III-ia–treated C6 glioma cells was measured by accumulated rodamine 123 (R123) fluorescence determined by flow cytometry. Data represent the mean ± SD (*P ≤ 0.05, **P ≤ 0.01 and ***P ≤ 0.001) of three independent experiments. **D)** cytosolic *cyt c* and activation of caspase 3 were determined by Western blot. The figure shown is representative of at least three different experiments for each experimental condition. E) Effect of ZVAD on Cas III-ia induced cytotoxicity in C6 glioma cells. It was determined by MTT assay in control cells and cells treated with Cas III-ia and Cas III-ia + 50μΜ ZVAD for 24 h; data represent the mean ± SD (*P ≤ 0.05, **P ≤ 0.01 and ***P ≤ 0.001) of three independent experiments.

To investigate the effect of Cas III-ia on apoptosis, drug-treated cells were loaded with TUNEL staining to identify apoptotic cells. Figure 4A shows the FITC-labeled fragmented DNA overlapping with the nuclear marker, DAPI. Most cells treated with Cas III-ia presented typical apoptotic morphology at all assayed doses**,** but a progressively stronger effect was obtained with rising drug concentrations (10, 15 and 20 μg/ml).

Mitochondria play an important role in the regulation of the apoptotic pathway, inducing a release of apoptotic mediators (cytochrome c, Smac/Diablo, Endo G and AIF) into the cytosol. This release is mediated by members of the Bcl-2 protein family which have either anti or proapoptotic functions [8]. For instance, the Bid pro-apoptotic protein, in response to an apoptotic signal, is cleaved by caspase 8 to give rise to the C- terminal product Bid_t_ (truncated Bid), which is myristolated and translocated to the mitochondria [29]. It has been proposed that Bid participates in the permeabilization of the outer mitochondrial membrane, and in the amplification of the pro-apoptotic signaling of Bax, either through direct interaction with Bax/Bak or by scavenging the anti-apoptotic Bcl-2 and Bcl-x_L_, which oppose Bax activity [30,31]. The possible participation of caspase 8, Bid and Bax in the antineoplastic effect induced by Cas III-ia on C6 glioma cells was examined by Western blot analysis. Figure 4B shows the activation of caspase 8, as well as the increment in Bid protein concentration and the cleavage of Bid to Bid_t_ induced by Cas III-ia at all assayed doses, as compared with controls. In addition, Bax content significantly increased at all assayed doses of Cas III-ia. These results indicate the participation of caspase 8, Bid_t_ and Bax in the antineoplastic effect of Cas III-ia on C6 glioma cells.

The fluorescent dye Rhod 123 internalizes inside energized mitochondria. To determine changes in mitochondrial functioning after Cas III-ia treatment, the mitocondrial membrane potential of C6 glioma cells loaded with Rhod 123 was measured. The quenching signal in Rhod-loaded cells is indicative of loss of membrane potential and, thus, of mitochondrial function. Changes in fluorescence were analyzed by flow cytometry. Cas III-ia treatment decreased the mitochondrial membrane potential by 26%, 30%, 54% and 71% at 5, 10, 15 and 20 μg/ml of Cas III-ia, respectively (Figure 4C).

The mitochondrial damage caused by Cas III-ia probably results in the release of *cyt c* into the cytosol and the activation of caspases. The presence of *cyt c* in the cytosol and activation of caspase 3 was determined by Western blot in C6 glioma cells exposed to Cas III-ia (Figure 4**D)**; significant release of *cyt c* into the cytosol was found at 10, 15 and 20 μg/ml of Cas III-ia when compared with controls and significant activation of caspase 3 at all doses of Cas III-ia. Addition of 50 μM Z-VAD-*FMK (*pan-caspase inhibitor) to Cas III-ia-treated cells provided modest protection from the Cas III-ia induced antineoplastic effect. These results suggest that apoptosis can be considered non-apoptotic cell death or caspase-independent cell death (Figure 4E) since the activity of caspase-3 was inhibited by Z-VAD-FMK in cells treated with Cas III-ia. This was determined by Western blot (Additional file 2: Figure S2**).**

### Intracellular ROS control autophagy and apoptosis induced by cas III-ia

**Figure 5.**
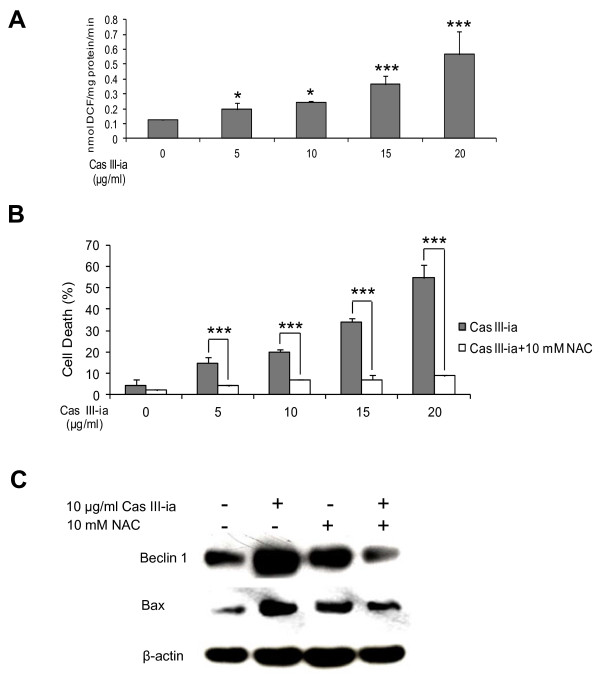
**Increase in cellular ROS is associated with autophagy and apoptosis induced by Cas III-ia. A)** ROS generation was determined in lysed cells obtained from controls and 24-h Cas III-ia-treated C6 cells as described in the Materials and Methods section. Each bar represents the mean ± SD (*P ≤ 0.05, **P ≤ 0.01 and ***P ≤ 0.001) of three independent experiments. **B)** Effect of NAC on cell death of 24 h Cas III-ia-treated cells in the presence of 10 mM NAC. For cell death assay, the cells were treated with 2.5, 5, 10, 15 and 20 μg/ml Cas III-ia for 25 h and analyzed by flow cytometry with propidium iodide. Data representative of means ± SD (*P ≤ 0.05, **P ≤ 0.01 and ***P ≤ 0.001) of three independent experiments. **C)** Expression of Beclin 1 and Bax proteins in lysated cells of control and treated cells with 10μg/ml Cas III-ia, 10mM NAC and Cas III-ia + 10 mM NAC were determined by W. blot. The figures shown are representative of at least three different experiments for each experimental condition.

The molecular mechanisms underlying the ability of Cas III-ia to simultaneously induce autophagy and apoptosis in C6 cells was investigated. First, intracellular ROS production generated by Cas III-ia was examined using the H_2_O_2_ sensitive fluorescent probe DCHF-DA. Results showed that incubation of cells with Cas III-ia resulted in significant increase of ROS production at all doses (Figure 5A). Pre-incubation of cells with the ROS scavenger N-acetyl-_L_- cysteine, significantly blocked cell death induced by Cas III-ia at all doses (Figure 5B). This finding indicates that ROS are involved in the cytotoxic effect induced by Cas III-ia. Interestingly, NAC also inhibited Bax and Beclin-1 expression induced by Cas III-ia (10 μg/ml) (Figure 5C). These results suggest that the presence of ROS may profoundly affect cellular response to apoptosis and autophagy.

### Cas III-ia induces the inactivation of antioxidant enzymes

**Figure 6:**
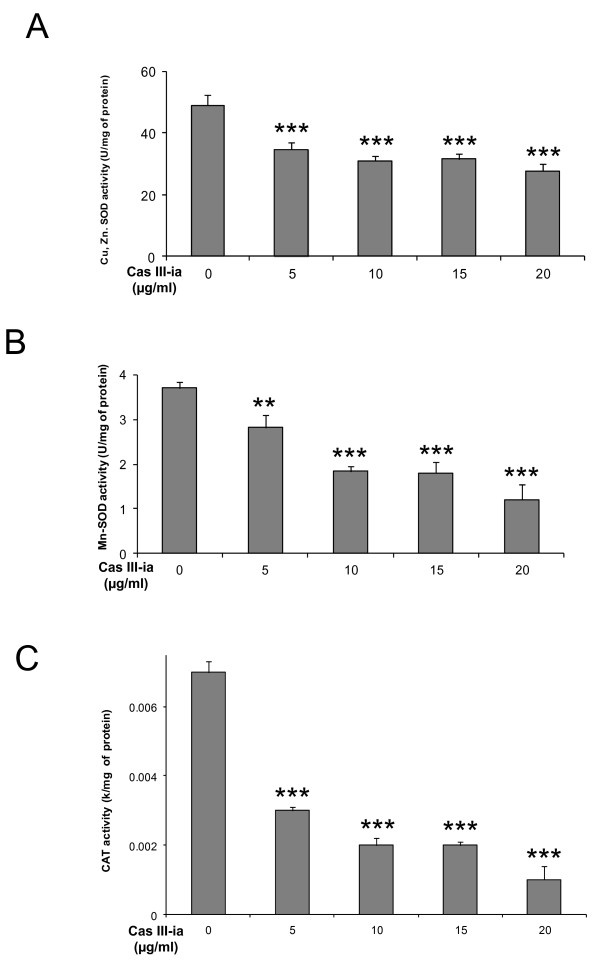
**Treatment with Cas III-ia induces a down in the enzymatic activation of SOD and CAT. A)** Enzymatic activity of Cu,Zn-SOD. **B)** Enzymatic activity of Mn-SOD. **C)** Enzymatic activity of CAT. The enzymatic activities were determined in lysated cells of control and treated cells with Cas III-ia for the techniques described in methods section. Data representative of means ± SD (*P ≤ 0.05, **P ≤ 0.01 and ***P ≤ 0.001) of three independent experiments.

Oxidative stress occurs as a consequence of the ROS burst. The decreasing antioxidant system could cause the accumulation of H_2_O_2_ or products of its decay and of O_2_. In this context, we measured the activity of two antioxidant enzyme types, SOD and catalase, involved in maintaining cellular redox balance, in the cellular lysates of glioma C6 cells treated with 5, 10, 15 and 20 μg/ml Cas III-ia for 24 h as well as in controls. Enzymatic activity of Cu/Zn-SOD decreased significantly in glioma C6 cells at all concentrations of Cas III-ia: treatment with 5, 10, 15 and 20 μg/ml Cas III-ia caused a fall in Cu/Zn enzymatic activity of 28%, 36%, 36% and 45% (34 ± 2, 31 ± 1, 31 ± 2 and 27 ± 2 U/mg protein), respectively, while the enzymatic activity in controls was 49 ± 3.4 U/mg protein (Figure 6A). Mn-SOD showed the same course with 25%, 50%, 50% 75% (3 ± 0.3, 2 ± 0.1, 2 ± 0.2 and 1 ± 0.3 U/mg protein) decrease, respectively; the enzymatic activity in controls being 4 ± 0.2 U/mg protein (Figure 6B). The same trend was found for catalase (CAT) activity, which decreased by 57%, 71%, 71% and 86% a (0.003 ± 0.0001, 0.002 ± 0.0002, 0.002 ± 0.0001 and 0.001 ± 0.0004 k/mg protein) at 5, 10, 15 and 10 μg/ml Cas III-ia, respectively, while enzymatic activity in controls was 0.007 ± 0.0003 k/mg protein (Figure 6C). These results suggest that one mechanism by which Cas III-ia induces ROS formation may be the inactivation of SOD and CAT. Cas III-ia-induced JNK activation determining the simultaneous induction of autophagy and apoptosis

**Figure 7:**
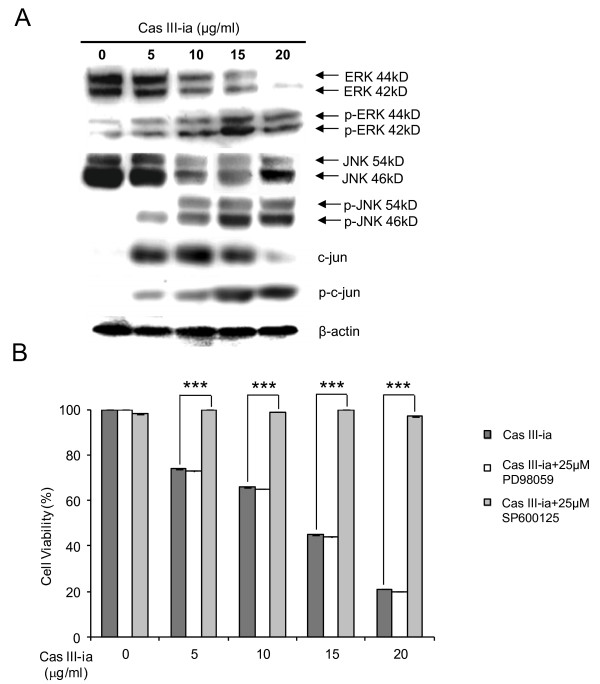
**Activation of JNK and ERK upon the exposure of Cas III-ia. A)** Total cell lysated from control cell and treated cells with Cas III-ia (5, 10, 15 and 20 μg/ml). were inmmuno-blot for detection of phospho-JNK, total JNK, phospho-ERK, total ERK, phosphor-c-jun and total c-jun. The figures shown are representative of at least three different experiments for each experimental condition. **B)** Effect of SP600125 (inhibitor de JNK) and PD98059 (inhibitor of ERK) on cell viability of 24 h Cas III-ia treated cells. The cell viability were determined by MTT assay in control cell and treated cells with Cas III-ia (5, 10, 15 and 20 μg/ml), Cas III-ia (5, 10, 15 and 20 μg/ml) + 25μM SP600125 and Cas III-ia (5, 10, 15 and 20 μg/ml) + 25μM PD98059. Data representative of means ± SD (*P ≤ 0.05, **P ≤ 0.01 and ***P ≤ 0.001) of three independent experiments.

To investigate the role of the MAPKs pathway in Cas III-ia induced cytotoxicity, the activation of JNK, ERK and p38 were studied by Western blot using phosphorylated antibodies which select the active form of these enzymes. We showed ERK and JNK activation, in a dose-dependent manner (Figure 7A). However, p38 was not activated (results not shown). One of the targets of JNK is c-jun, a member of the AP-1 transcription factor. We determined both, total c-jun and pc-jun by Western blot. Figure 7A shows that the contents of p-c-jun increased in a dose-dependent manner by Cas III-ia treatment.

In addition, JNK activation was determined at 6, 12 and 24 h in cell lysate from cells treated with 10 μg/ml of Cas III-ia and controls. (Additional file 3: Figure S3), the figure shows activation of JNK from 6 h to 24 h of treatment.

To determine the involvement the MAPKs, we investigated the effects of pharmacological inhibitors of JNK and ERK. Cells were pre-incubated with or without SP600125 (25μM) or PD98059 (25μM) during 1 h, followed by Cas III-ia treatment for 24 h. The JNK specific kinase inhibitor SP600125 hindered the cytotoxic effects induced by Cas III-ia at all doses; while PD98059 did not inhibit the cytotoxic effects induced by Cas III-ia (Figure 7B). These results suggest that JNK activation may be an important requirement by Cas III-ia-induced cell death, and that the participation of ERK is not critical.

### ROS induce JNK activation

**Figure 8:**
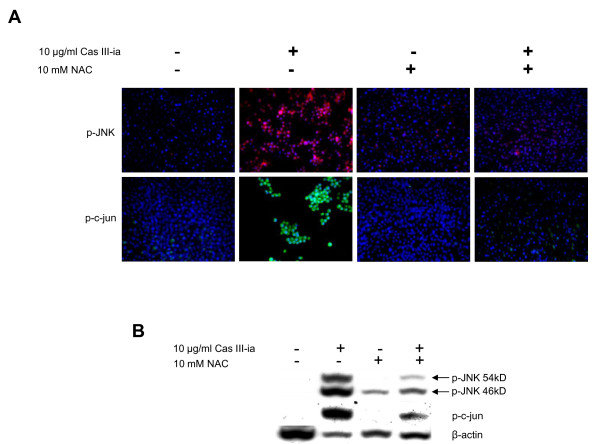
**ROS induce JNK activation. A)** Inmunocytochemistry of phospho-JNK (red- rodamine and blue–DAPI) and phospho-c-jun (Green-FITC and blue-DAPI) in control cells, treated with 10 μg/ml Cas III-ia and 10 μg/ml Cas III-ia + 10mM *N*-acety-L- cysteine (NAC) for 24 h. The figures shown are representative of at least three different experiments for each experimental condition. Original magnification, x20. **B)** Western blot of phospho-JNK and phospho-c-jun in controls, cells treated with 10 μg/ml Cas III-ia and 10 μg/ml Cas III-ia + 10mM NAC for 24 h. The figures shown are representative of at least three different experiments for each experimental condition.

To determine whether Cas III-ia-induced ROS led to activation of JNK in malignant glial cells, we determined the expression of pJNK and pc-jun by immunocytochemistry and Western blot in non-treated cells and in cells pre-incubated for 1 h with or without *N*-acety-L- cysteine (NAC), followed by treatment with 10 μg/ml Cas III-ia for 24 h. Figure 8A and 8B show that the activation of JNK and c-jun induced by Cas III-ia were significantly diminished by NAC. These results suggest that ROS production increased by Cas III-ia contributes, at least partially, to the activation of JNK, and that ROS is upstream of JNK.

## Discussion

Autophagy has emerged as a powerful mediator of programmed cell death, either opposing or enhancing apoptosis, or acting as an alternative form of programmed cell death, different from apoptosis [14]. The present study shows that Cas III-ia induces cell death by both autophagy and apoptosis in rat C6 glioma cells. A microscopic analysis of cultured cells 24 h after Cas III-ia administration revealed a significant number of cells showing coexistence of both apoptosis (cell shrinkage, margination and chromatin condensation) and autophagy (autophagic vacuoles and autophagosomes).

Beclin 1 is the mammalian orthologue of the yeast Vps30/Apg6 gene, required for autophagosome formation, and is monoallelically deleted in a high percentage of human carcinomas [28]. In MCF7 breast carcinoma cells the expression of Beclin 1 protein decreases below detectable levels. Stable transfection of Beclin 1 in MCF7 cells promotes autophagy and reduces tumorigenic capacity, suggesting that autophagic activity is associated with the inhibition of cell proliferation [32]. Tamoxifen, a drug used to treat breast cancer, may function by activating autophagy, possibly by upregulating Beclin1 in a process mediated by ceramide [33]. In this study, we observed the inhibition of cell viability and overexpression of the Beclin 1 protein in C6 glioma cells after Cas III-ia treatment. Our results suggest that upregulation of Beclin 1 may contribute to the antineoplastic effect of Cas III-ia.

Recent studies have shown that LC-3, a modifier protein, is processed by a unique protein activation/conjugation system, to form autophagosomal membranes during autophagy; where LC-3 becomes associated with an autophagosomal precursor to form a cup-shaped pre-autophagosome, which finally closes to form autophagosomes that engulf the cytosolic compartment, the autophagosomes fuse with lysosomes to form autolysosomes [34]. Present results show LC-3-II formation induced by Cas III-ia in glioma C6 cells, by a mechanism which is not yet clearly understood.

LTR is an acidotropic fluorescent probe used to label and track acidic organelles in living cells, including lysosomes, autophagosomes, late endosomes and, to a lesser extent, early endosomes less acidic than other organelles [21]. An increment in LTR-flouresence represents an increase in autophagosomes and autolysosomes [21]. In our study, we observed by confocal microscopy a significant increase in the size and number of lysosomal/autophagosomal compartments in response to all doses of Cas III-ia, as compared with controls.

PI3K is a conserved family of lipid kinases that catalyze the phosphorylation of position 3 on the inositol ring of phosphoinositides [35]. They produce lipids involved in cell proliferation, differentiation, apoptosis, autophagy, cytoskeletal organization, and membrane trafficking. The drug 3-MA commonly used to inhibit the autophagic pathway [36] interferes with the activity of class III PI3K by interrupting autophagy at the sequestration step [35,37]. In our study, 3-MA enhanced cell death induced by Cas III-ia in malignant glioma cells. It seems that autophagy induced by Cas III-ia may antagonize or delay apoptosis; thus, inhibition of autophagy by 3-MA may increase the sensitivity of the cell to cell death signals. Similarly, it has been shown that the inhibition of N-(4- hydroxyphenyl) retinamide-induced autophagy enhances cell death in malignant glioma cells [38]. Further studies have suggested that inhibition of autophagy induced by radiation/arsenic trioxide/temozolamide decreases survival of glioma cells [28,39,40] and that autophagy antagonizes cell death [41-43]. Our results suggest that inhibition of autophagy prevents the removal of damaged mitochondria, promoting loss of ∆Ψm and subsequent ROS generation, thereby accelerating cell death. When autophagy is inhibited, enhanced cell death may be coupled to an increase in mitochondrial depolarization and ROS generation, thereby increasing mitochondrial damage, and leading to the release of cell death-inducing molecules [38,41,42] such as cyt c, SMAC/Diablo and AIF, which then activate the caspase-dependent or-independent apoptotic pathways.

This work also investigated if Cas III-ia induces apoptosis of C6 glioma cells. Tunnel assay results showed that Cas III-ia induced apoptosis, with most of the cells positive at 10, 15 and 20 μg/ml, and a slight decrease in cell viability at 5 and 10 μg/ml of Cas III-ia, determined by mitochondrial activity and mitochondrial membrane potential. One possible explanation of these results is that the TUNEL assay is not specific for cell death by apoptosis, since it may stain both apoptotic and autophagic cells [44]. It has been reported that autophagy induced by 4-HPR is associated to slow loss of ∆Ψm, while apoptosis is associated to rapid loss of ∆Ψm [38]. Another possible explanation of the decrease in mitochondrial activity and mitochondrial membrane potential at 5 and 10 μg/ml of Cas III-ia is that Cas III-ia may initiate apoptosis by an extrinsic pathway and subsequently activate an intrinsic pathway, since Cas III-ia induces the activation of caspase 8 (the initiating caspase via death receptors) and capase 3, formation of Bidt and Bax; all of these markers initiating at low concentrations. On the other hand, a pronounced fall in mitochondrial membrane potential was detected, and a release of cytosolic cyt-c at high concentrations.

However, abrogation of caspase activation did not prevent cell death; suggesting that the antineoplastic effect of Cas III-ia can be considered as non-apoptotic cell death or caspase-independent cell death. In a previous report we showed that another Casiopeina, the Cas IIgly [Cu(4,7-dimethyl-1,10-phenanthroline)(glycine)(H_2_O)]NO_3_, may induce apoptosis in CH1 cells, with no evidence of DNA laddering and independent of caspase activation [5]. In C6 glioma cells, Cas IIgly also induces apoptosis by a caspase-independent mechanism, mediated by apoptosis induction factor (AIF) and endonuclease G [45]. Our findings suggest that Cas III-ia induces autophagy and apoptosis, both processes being caspase-activation independent. Similarly, TNFα, a member of the apoptosis-inducing family, stimulates autophagy and apoptosis of T-lymphoblastic leukemia cells, independent of caspases [46]**.**

These results show that neither selective pharmacological inhibition of apoptosis nor of autophagy prevented the antineoplastic effects on glioma cells induced by Cas III-ia, suggesting that both pathways are essential in the cell death process.

Previous studies have shown that ROS may serve as signaling molecules that directly or indirectly activate both autophagy and apoptosis. Overexpression of TrkA diminishes catalase activity, leading to the accumulation of ROS and subsequent autophagy and apoptosis [47]. Moreover, under starving conditions, ROS oxide cysteine residues of Atg4, induce autophagy and activate the transcription of autophagy-related genes, such as Beclin1 [19,48]. In our study, Cas III-ia induced ROS generation, and reduced SOD1, SOD2 and catalase activity. Pretreatment with *N*-acety-L- cysteine (NAC) showed a protective effect on Cas III-ia induced cell death. Moreover, overexpression of Beclin 1 and Bax induced by Cas III-ia were almost completely inhibited by NAC, suggesting that Cas III-ia induces autophagy and apoptosis by the generation of ROS.

**Figure 9:**
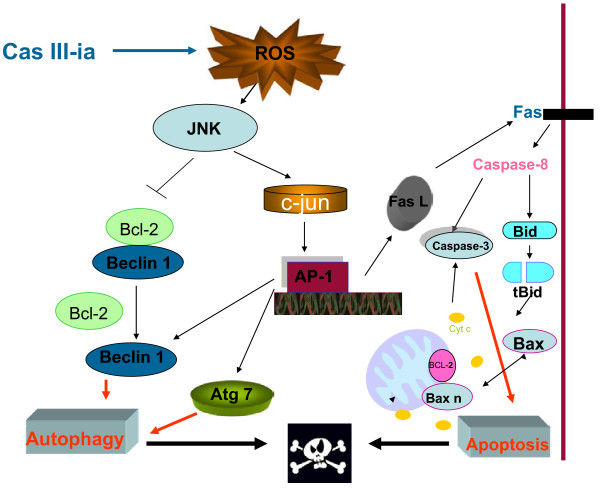
**Suggested pathway initiated by Cas III-ia leading to autophagy and apoptosis in C6 glioma cells.** Cas III-ia may cause oxidative stress by JNK activation. JNK can phosphorylate Bcl-2 and release Beclin 1 enhancing autophagy. Alternatively, JNK can induce phosphorylation of c-jun; phospho-c-jun favors the production of AP-1 which, in turn, increases the expression of many genes among which are c-jun itself, Beclin, Atg7, Bax and FasL. Beclin 1 and Atg 7 may induce autophagy and FasL can stimulate Fas-R inducing the activation of caspase 8, which then degrades Bid into Bidt inducing Bax oligomerization and the depolarization of the mitochondrial membrane with release of cyt c and the consequent activation of caspase 3. Autophagy and apoptosis can thus induce cell death.

Various stimuli activate JNK, which participates in the regulation of fundamental cellular pathways such as autophagy and apoptosis [49]. JNK phosphorylates several proteins of the Bcl-2 family, resulting in inhibition of the antiapoptotic activity of Bcl-2 and Bcl-x_L,_ and the activation of Bax [50]. Interestingly, it has been shown that the activation of JNK results in the phosphorylation of Bcl-2 which enhances autophagy and cell survival by disrupting the interaction between Bcl-2 and Beclin1, while prolonged Bcl-2 phosphorylation mediated by JNK promotes apoptosis [51]. Futhermore, as JNK phosphorylates the c-jun transcription factor it promotes the upregulation of autophagic and apoptotic genes, such as Beclin 1 and Fas [52]; also, JNK induces the expression of Atg7, a crucial mediator of autophagosome formation [48]. In agreement with these findings, we demonstrated that Cas III-ia induces JNK activation, phosphorylation of c-jun and expression of Beclin 1, Atg 7 and Bax. Pharmacological inhibition of JNK prevented the antineoplastic effect of Cas III-ia. We also found that ROS generation mediates the activation of JNK in the pathway of Cas III-ia –induced cell death (Figure 9).

Most antineoplasic drugs against glioma are highly toxic and have limited efficacy, as they also affect normal cells. Lipopholic cation drugs concentrate into mitochondria due to their negative electric membrane potential; the higher plasma and mitochondrial membrane potentials of tumor cells may enhance the selective targeting by Cas III-ia of tumor cells, particulary inside mitochondria. Such is the case of AS-30D hepatoma mitochondria, which exhibit higher mitochondrial membrane potential values than those from normal hepatocytes. Indeed, AS-30D and HCT-40 cells in culture selectively die within 48 h of exposure to Cas III-ia, co-cultured normal fibroblasts survive [7,53] the effect of Cas III-ia (5, 10, 15, and 20 μg/ml). In these experiments, at a 5-10 μg/ml dose of Cas III-ia, cell viability was 100%; when the dose was increased to 15 μg/ml, viability was 90%, and at 20 μg/ml, it fell to 83%, suggesting that the metabolic effect of Cas III-ia at 5-10 μg/ml doses is fairly specific against malignant cells.

## Conclusions

Our observations show that Cas III-ia promotes accumulation of intracellular ROS, resulting in sustained activation of JNK, which in turn leads to autophagy and apoptosis of C6 glioma cells.

Taken together, present data stress the potential of this copper compound in the therapeutic induction of cell death of susceptible tumor cells responsive to autophagic or apoptosis stimuli mediated by ROS induction and JNK activation.

## Abbreviations

Cas III-ia, Casiopeina III-ia; LC3, Light chain 3; ROS, Intracellular reactive oxygen species; JNK, C-jun NH2-terminal kinase (JNK); ERK, Extracellular signal-regulated kinase; 3-MA, 3-Methyladenine; Z-VAD-FMK, Benzxycarbonyl-Val-Ala-Asp Z-VAD; NAC, N-acety-L- cystein; MTT, 3[4,5-dimethylthiazol-2-yl]-2,5-diphenyl-tetrazolium bromide; Rhod 123, Rhodamine 123; LTR, LysoTracker Red; DCFH-DA, 2',7'-dichlorofluorescein diacetate.

## Competing interests

The authors declare that they have no competing interests.

## Authors´ contributions

CTS, DJF, SRE, AC, JS designed and performed the research, analyzed the data and drafted the manuscript; FFV performed the confocal and electronic microscopy studies; LRA designed the Casiopeina. All authors have read and approved the final manuscript.

## Pre-publication history

The pre-publication history for this paper can be accessed here:

http://www.biomedcentral.com/1471-2407/12/156/prepub

## Supplementary Material

Additional file 1: Figure S1 **3-MA inhibits cell death induced by TMZ.** The effects of 3-MA on TMZ induced cytotoxicity in C6 glioma cells were determined by the MTT assay in control cells and cells treated with TMZ and TMZ + 5 mM 3-MA for 24 h; data represent the mean ± SD (*P ≤ 0.05, **P ≤ 0.01 and ***P≤ 0.001) of three independent experiments. Click here for file

Additional file 2: Figure S2 **Effect of ZVAD on caspase 3 activity.** Caspase 3 activity was determined by Western blot in control cells and cells treated with Cas III-ia and Cas III-ia + 50μΜ ZVAD for 24 h. The figures shown are representative of at least three different experiments for each experimental condition. Click here for file

Additional file 3: Figure S3 **Persistent activation of JNK upon exposure to Cas III-ia.** Total cell lysate from control cells and cells treated with 10 μg/ml Cas III-ia for 6, 12 and 24 h were inmmunoblotted to detect phospho-JNK and total JNK. The figures shown are representative of at least three different experiments for each experimental condition. Click here for file
