# Supplementary figures and images for: Copper compound induces autophagy and apoptosis of glioma cells by reactive oxygen species and jnk activation
Source: BMC Cancer. 2012 Apr 27;12:156. doi: 10.1186/1471-2407-12-156 (PMC3404907; doi:10.1186/1471-2407-12-156)

## Slide 1
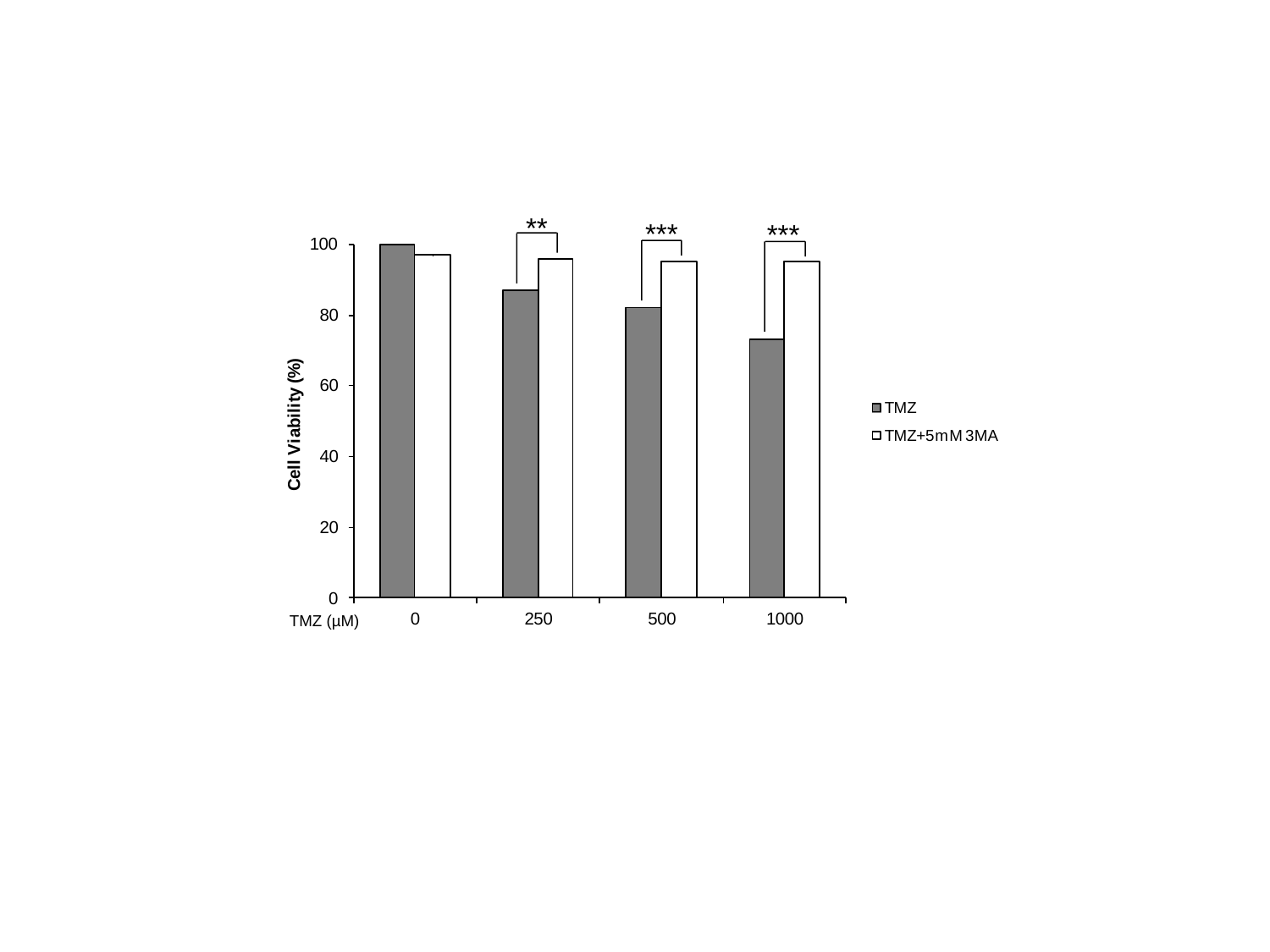

**
***
***
TMZ (µM)

Supplement: Additional file 1: Figure S1 — 3-MA inhibits cell death induced by TMZ. The effects of 3-MA on TMZ induced cytotoxicity in C6 glioma cells were determined by the MTT assay in control cells and cells treated with TMZ and TMZ + 5 mM 3-MA for 24 h; data represent the mean ± SD (*P ≤ 0.05, **P ≤ 0.01 and ***P≤ 0.001) of three independent experiments. [file 1471-2407-12-156-S1.ppt]

## Slide 1
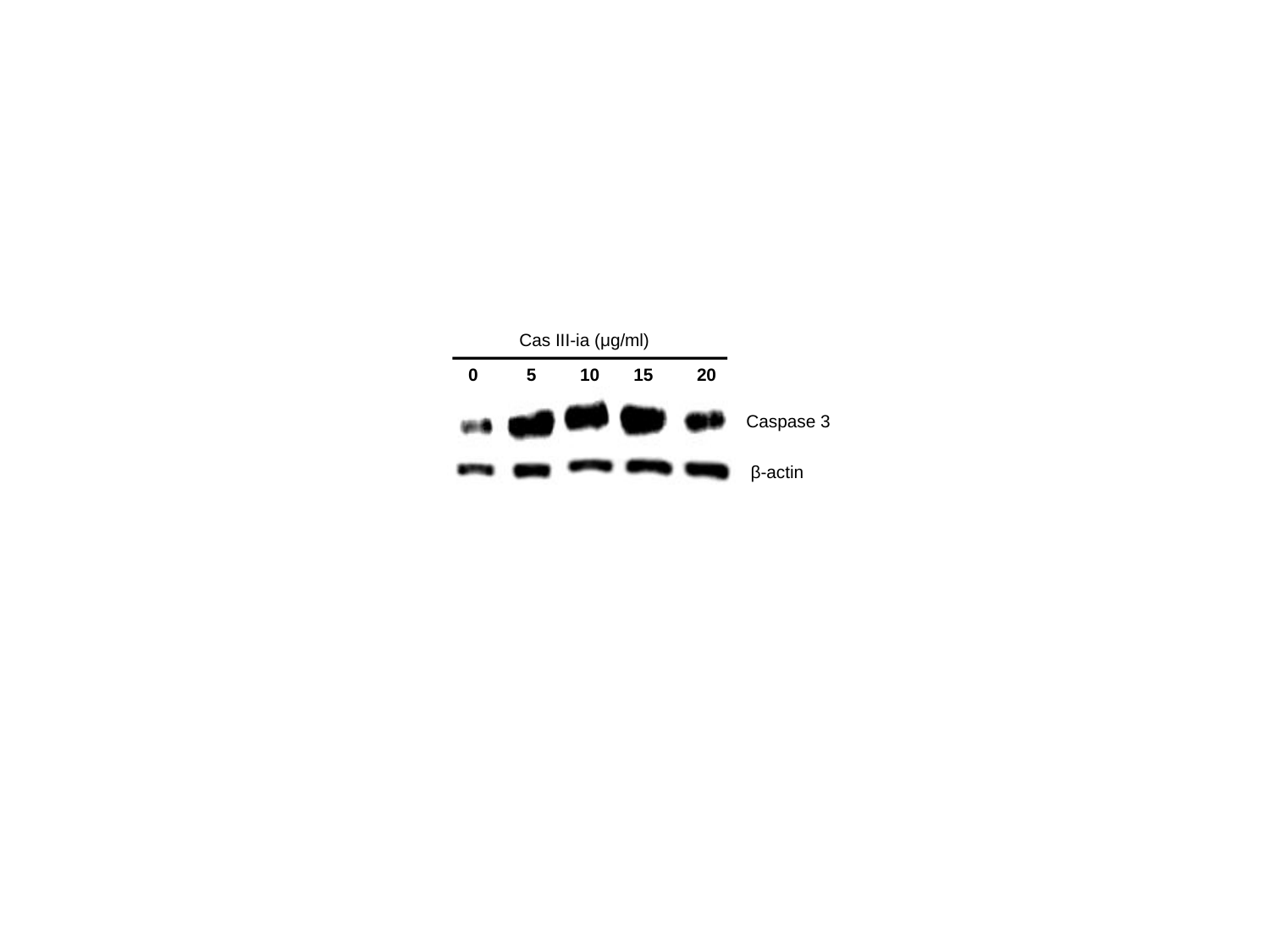

Cas III-ia (μg/ml)
 0 5 10 15 20
Caspase 3
β-actin

Supplement: Additional file 2: Figure S2 — Effect of ZVAD on caspase 3 activity. Caspase 3 activity was determined by Western blot in control cells and cells treated with Cas III-ia and Cas III-ia + 50μΜ ZVAD for 24 h. The figures shown are representative of at least three different experiments for each experimental condition. [file 1471-2407-12-156-S2.ppt]

## Slide 1
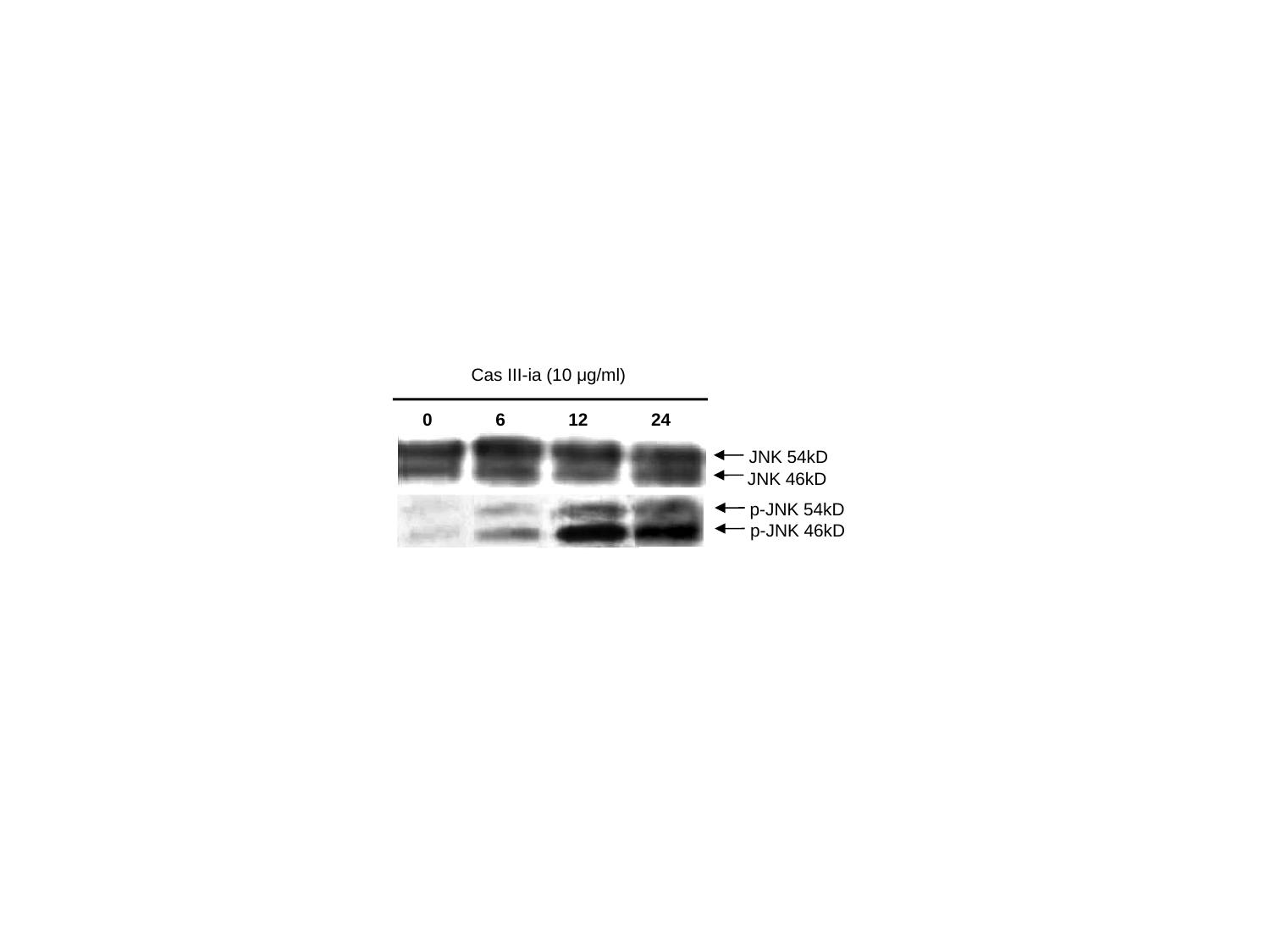

Cas III-ia (10 μg/ml)
0 6 12 24
JNK 54kD
JNK 46kD
p-JNK 54kD
 p-JNK 46kD

Supplement: Additional file 3: Figure S3 — Persistent activation of JNK upon exposure to Cas III-ia. Total cell lysate from control cells and cells treated with 10 μg/ml Cas III-ia for 6, 12 and 24 h were inmmunoblotted to detect phospho-JNK and total JNK. The figures shown are representative of at least three different experiments for each experimental condition. [file 1471-2407-12-156-S3.ppt]
